# Can pharmacogenetics impact the therapeutic effect of cytarabine and anthracyclines in adult acute myeloid leukaemia patients?: A Serbian experience

**DOI:** 10.5937/jomb0-47459

**Published:** 2024-06-15

**Authors:** Zlatko Pravdić, Nada Suvajdžić-Vuković, Marijana Virijević, Mirjana Mitrović, Nikola Pantić, Nikica Sabljić, Đorđe Pavlović, Irena Marjanović, Zoran Bukumirić, Ana Vidović, Ljubomir Jaković, Sonja Pavlović, Vladimir Gašić

**Affiliations:** 1 University Clinical Centre of Serbia, Clinic of Haematology, Belgrade; 2 University of Belgrade, Faculty of Medicine, Belgrade; 3 University of Belgrade, Institute of Molecular Genetics and Genetical Engineering, Belgrade; 4 University of Belgrade, Faculty of Medicine, Institute of Medical Statistics and Informatics, Belgrade

**Keywords:** AML, anthracyclines, cytarabine, pharmacogenetic variants, AML, antraciklini, citarabin, farmakogenetičke varijante

## Abstract

**Background:**

Cytarabine-anthracycline-based induction chemotherapy remains the standard of care for remission induction among patients with newly diagnosed acute myeloid leukaemia (AML). There are remarkable differences in therapy response among AML patients. This fact could be partly explained by the patients' genetic variability related to the metabolic paths of cytarabine and anthracyclines. This study aims to evaluate the effect of variants in pharmacogenes SLC29A1, DCK, ABCB1, GSTM1, and GSTT1, as well as laboratory and AML-related parameters on clinical outcomes in adult AML patients.

**Methods:**

A total of 100 AML patients were included in the study. Pharmacogenetic variants SLC29A1 rs9394992, DCK rs12648166, ABCB1 rs2032582, and GSTM1 and GSTT1 gene deletions were detected by methodology based on PCR, fragment analysis and direct sequencing. The methods of descriptive and analytic statistics were used. Survival analysis was done using the Kaplan-Meier method using the Log-Rank test.

**Results:**

This is the first study of adult AML pharmacogenetics in the Serbian population. Clinical outcomes in our cohort of AML patients were not impacted by analysed variants in SLC29A1, DCK, ABCB1 and GSTT1, and GSTM1 genes, independently or in combinations. Achievement of complete remission was identified as an independent prognostic indicator of clinical outcome.

**Conclusions:**

The population-specific genomic profile has to be considered in pharmacogenetics. Since the data on AML pharmacogenetics in European populations is limited, our results contribute to knowledge in this field and strongly indicate that a high-throughput approach must be applied to find particular pharmacogenetic markers of AML in the European population.

## Abstract (sr)

**Uvod:**

Indukciona terapija zasnovana na citarabinu i antraciklinu standard je lečenja novodijagnostikovanih odraslih pacijenata sa akutnom mijeloidnom leukemijom (AML). Ishodi lečenja među obolelima od AML značajno se razlikuju. Ove razlike bi se delimično mogle objasniti genetičkim varijabilitetom metaboličkih puteva citarabina i antraciklina. Cilj ovog istraživanja bilo je ispitivanje uticaja varijanti u farmakogenima SLC29A1, DCK, ABCB1, GSTM1 i GSTT1, kao i laboratorijskih i parametara vezanih za AML na ishode lečenja odraslih bolesnika sa AML.

**Metode:**

Ukupno 100 bolesnika sa AML je uključeno u studiju. Farmakogenetičke varijante SLC29A1 rs9394992, DCK rs12648166, ABCB1 rs2032582 i delecije gena GSTM1 i GSTT1 određivane su metodologijom zasnovanom na PCR-u, analizom fragmenata i direktnim sekvenciranjem. Korišćene su metode deskriptivne i analitičke statistike. Analiza preživljavanja je sprovedena prema Kaplan-Majerovom metodu upotrebom Log-Rank testa.

**Rezultati:**

Ovo je prva farmakogenetička studija odraslih bolesnika sa AML u srpskoj populaciji. Varijante u genima SLC29A1, DCK, ABCB1, GSTT1 i GSTM1 nisu uticale na ishode lečenja u našoj kohorti obolelih od AML, samostalno ili u međusobnim kombinacijama. Međutim, postizanje kompletne remisije bolesti istaklo se kao nezavisni prediktor ishoda lečenja.

**Zaključak:**

Prilikom farmakogenetičkih istraživanja neophodno je razmotriti jedinstveni genetički profil ispitivane populacije. Kako su farmakogenetički podaci o AML u evropskim populacijama oskudni, naši rezultati doprinose proširenju saznanja u ovoj oblasti i ukazuju na značaj primena tehnika sekvenciranja nove generacije u cilju otkrivanja posebnih farmakogenetičkih markera kod obolelih od AML u evropskim populacijama.

## Introduction

The main treatment for most types of AML is chemotherapy (ChT), sometimes along with a targeted therapy drug. The standard ChT for AML is nucleoside analogue cytarabine and anthracyclines (daunorubicin, idarubicin) combined in the ChT regimen »7+3« [1]. Despite high remission rates up to 60–80%, the 5-year overall survival (OS) is <50% and <20% for < 60-year-old and 60-year-old patients, respectively [2] [3]. This grim clinical outcome could be partly explained by the patients’ genetic variability, which impacts the proteins involved in the metabolic paths of cytarabine and anthracyclines.

In standard induction doses of 100–200 mg/m^2^ IV for seven days in »7+3 regimen«, with plasma concentrations <1 μmol/L, cytarabine primarily enters the leukemic cells by nucleoside transporter SLC29A1 (solute carrier family 29 member 1) [2] [4]. However, in the higher doses of 1–2 g/m^2^ IV, given in the consolidation cycles when it reaches the plasma concentrations 10 μmol/L, cytarabine transport is independent of nucleoside transporters and diffuses freely into the leukemic cells [2] [4]. Intracellularly, cytarabine is activated in a stepwise process to cytarabine triphosphate. The first and rate-limiting process is mediated by deoxycytidine kinase (DCK). Cytarabine antileukemic effect is based on inhibiting the conversion of citidilate to 2’deoxycitidilate (essential for DNA synthesis), by incorporating DNA and RNA molecules and inhibiting DNA-dependent polymerase [3]. Single nucleotide polymorphisms (SNPs) in *SLC29A1* rs9394992 and *DCK* rs12648166 are associated with the risk of development and the clinical outcome in other malignancies [4]. Referring to the importance of these genes in cytarabine action, these variants could be regarded as potential pharmacogenetic markers in AML.

Anthracyclines exert their antileukemic effect by intercalating the DNA helix, disrupting DNA replication by inhibiting topoisomerase II, and producing reactive oxygen species (ROS) [3]. So far, potential pharmacogenetic candidate markers for anthracyclines could be genes coding anthracycline efflux pumps–ATP-binding cassettes (ABC), influencing the intracellular anthracycline concentration, or the genes coding intracellular ROS detoxifiers glutathione S-transferases (GST), affecting the extent of anthracycline- induced oxidative stress. *ABCB1*, coding p-glycoprotein, is one of the most commonly evaluated genes in AML. SNP of *ABCB1*rs20132582, c.2677G>T/A showed that minor alleles are correlated with decreased pump function [5] and subsequent higher toxicity and a higher CR rate [6]. Cytosolic GST family consists of seven classes, primarily evaluated alpha, mi, phi, and theta (A, M, P, T, respectively). Besides, deleterious (null) genotypes of *GSTM1* and *GSTT1* with consequent null enzymatic activity are associated with increased toxicity, lower CR rate, and decreased OS in AML patients treated with anthracyclines [5].

So far, the best pre-treatment predictors of outcome regarding CR rate, disease-free survival (DFS), and OS in adult AML patients are karyotypic and molecular abnormalities (e.g., *fms-related receptor tyrosine kinase 3 inter tandem duplication (FLT3-ITD)* and nucleophosmin 1 (*NPM1*)) [3] incorporated in 2022 European LeukemiaNet (ELN 2022) risk stratifications system for AML [1].

This study aims to evaluate the effect of variants in pharmacogenes *SLC29A1, DCK, ABCB1, GSTM1* and *GSTT1*, as well as laboratory and AML-related parameters on clinical outcome in adult AML patients.

## Materials and methods

The study was approved by the Ethics Committee of the University Clinical Centre of Serbia (UCCS) (N^0^ 1435/10). A total of 100 newly-diagnosed consenting Serbian adults aged 18–62 years, diagnosed with AML, except acute promyelocytic leukaemia, in the Clinic of Haematology UCCS from January 2015 to January 2018, were included in a retrospective cohort study. Additional inclusion criteria were Eastern Cooperative Oncology Group, Performance Status (ECOG PS) 2 and Hematopoietic cell transplantation – specific comorbidity index (HCT-CI score) <3 [7] [8]. According to ELN recommendations [9] patients received one or two inductions »7+3« cycles (cytarabine 100–200 mg/m^2^ IV, days 1–7 and daunorubicin 45–60 mg/m^2^ IV days 1–3). In patients achieving CR consolidation, treatment was undertaken either with three cycles of cytarabine (3000–6000 mg/m^2^ IV, days 1–3) or with allogenic hematopoietic stem cell transplantation (HSCT) in selected patients.

Demographic, standard laboratory, and AML-related parameters were collected from patients’ health records. These include age, gender, clinical presentation (enlarged lymph nodes, hepatosplenomegaly, bleeding, gingival hyperplasia, infiltration of central nervous system), complete blood count with differential, complete biochemistry parameters including serum lactate dehydrogenase (LDH), blood/ bone marrow blast percentage, cytologic, flow cytometry and genetic markers, enabling ELN 2022 risk stratification, treatments – induction, second induction, consolidation and a number of consolidations, HSCT, eventual post-relapse therapy and measurable residual disease (MRD). Standard flow cytometry markers were detected (CD45, TdT, CD34, HLA-DR, CD19, CD20, CD22, CD79a, CD10, CD2, CD3, CD5, CD7, CD13, CD33, CD117, CD15, CD14, CD4, CD64, CD36, Glycophorin A, CD41, CD42, CD61) [9] [10]. Marker positivity was defined by the presence of 20% on leukemic cells. Classic cytogenetic analyses [11] and detection of *FLT3-ITD* and *NPM1* mutations were performed [12] [13] [14].

Variants of *SLC29A1* rs9394992, *DCK* rs12648166, *ABCB1* rs2032582, and *GSTM1* and *GSTT1* gene deletions were detected by using methodology based on PCR, fragment analysis and direct sequencing of the pre-treatment bio-banked bone marrow aspirates and buccal swabs, analysed in the Laboratory for molecular biomedicine of the Institute for Molecular Genetic and Genetic Engineering. DNA was isolated by QIAamp DNA Blood Mini Kit (Qiagen, Germany). The *SLC29A1* variant was detected by allelic discrimination [15], whereas the DCK variant was detected using PCR and direct sequencing [16]. According to the manufacturer’s instruction, the *ABCB1* variant was genotyped by competitive allele-specific PCR-genotyping system (KASP) (LGC, Teddington, Middlesex, UK). *GSTM1* and *GSTT1* homozygous deletions (null genotype) were detected using PCR [17].

Complete response (CR), relapse, refractory disease, and clinical outcome measures – early death (ED), DFS, and OS were evaluated using ELN 2022 criteria [1]. Regarding CR, the number of induction courses to obtain CR and the time required to achieve it were reported. In regard to ED, the day after admission and cause of death was reported. In addition, time to relapse and post-relapse therapy were reported. MRD based on the leukaemia-associated immunophenotypes (LAIPs) was assessed using multiparameter flow cytometry [18]. DFS was calculated from the day of CR achievement to the relapse, death, or last visit, while OS was defined as the duration from the date of diagnosis until death or last visit [1].

The methods of descriptive statistics (mean with range and relative numbers) and analytic statistics (Mann-Whitney U Test, Chi-square test, and Fisher test of exact probability) were used. Correlations were tested using the Spearman rank coefficient, whereas survival analysis was done using the Kaplan-Meier method and the Log-Rank test. Logistic regression, uni- and multivariant Cox proportional-hazards regression models was used to identify prognostic factors associated with CR, relapse, ED, refractory disease, DFS, and OS. The influence of gene variants on clinical outcomes was tested in a dominant, recessive, and codominant manner. The combined effect of *SLC29A1*r s9394992, *DCK* rs12648166, *ABCB1* rs2032582 in dominant and *SLC29A1* rs9394992 and *DCK* rs12648166 in recessive manner was also tested. Statistical analysis was performed on SPSS software v20 (IBM corporation).

## Results

Baseline patient characteristics, treatment patterns, and outcomes are represented in Table 1 and Table 2. In our group of 100 Serbian patients with a median age of 51 years (range: 18–62), the male-to-female ratio was 52/48, respectively. Fifty-one patients had normal karyotype, *FLT3-ITD* and *NPM1* were detected in 14 and 16 patients, respectively. According to ELN 2022, 16, 60, and 24 patients were classified into favourable, intermediate, and unfavourable cytogenetic risk categories, respectively. CD34 and CD25 – positivity were registered in 57 and 2 patients, respectively. Eighty percent of patients received standard dose induction (cytarabine 200 mg/m^2^ IV for seven days and daunorubicin 60 mg/m^2^ IV for three days), while in 20% of older patients, according to ELN recommendations [9], a reduced dose of ChT (cytarabine 100 mg/m^2^ and daunorubicin 45 mg/m^2^) was administered. The second »7+3« cycle was administered in 37 patients. CR was achieved in 57 (57%) patients. ED occurred in 19 of the patients, with the causes shown in Table 2. A total of 17 (17%) patients had primary refractory disease. Consolidation was given in 57 (57%) patients – one, two and three cycles of intermediate/high doses of cytarabine were administered in 7 (12%), 15 (26%) and 35 (62%), respectively. A total of 30 patients underwent HSCT. A total of 33 patients relapsed. Median DFS was 7.3 months (range: 0.3–88.2), while median OS was 9 months (range: 0.3–88.2).

**Table 1:** Baseline patient characteristics. Abbreviations: CNS, central nervous system; ELN, European LeukemiaNet.

Variable	Number (%) <br>n=100 (100%)	Median <br>(range)
Baseline patient characteristics		
Age		51 (18–62)
Age>50/>55 years	53 (53%)/36 (36%)	
Gender, male/female	52 (52%)/48 (48%)	
Enlarged lymph nodes on diagnosis, present	19 (19%)	
Hepatosplenomegaly on diagnosis, present	38 (38%)	
Bleeding on diagnosis, present	37 (37%)	
Gingival hyperplasia, present	8 (8%)	
CNS infiltration, present/out of tested	15/40 (37.5%)	
Leucocytes x10^9^/L on diagnosis		16.5 (1–349)
Leucocytes>30x10^9^/L	31 (31%)	
Haemoglobin g/L on diagnosis		98 (65–166)
Platelets x10^9^/L on diagnosis		43.5 (1–422)
Lactate dehydrogenase on diagnosis U/L		253 (2–4169)
Lactate dehydrogenase >450 U/L	36 (36%)	
% of bone marrow/peripheral blood blasts		61.5 (20–97)/16 (0–98)
Karyotype: normal/abnormal	51/100 (51%)	
Cytogenetic risk (ELN 2022)		
Favorable/Intermediate/ Unfavorable	16 (16%)/60 (60%), 24 (24%)	
FLT3-ITD+	14 (14%)	
NPM1 mutated	16 (16%)	
CD34+	57 (57%)	
CD25+	2 (2%)	

**Table 2:** Treatment, clinical outcomes, and survival.

Variable	Number (%) <br>n=100 (100%)	Median (range)
Treatment		
Induction therapy »7+3«		
cytarabine 200 mg/m^2^, DA 60 mg/m^2^	80 (80%)	
cytarabine 100 mg/m^2^, DA 45 mg/m^2^	20 (20%)	
Second induction 7+3, yes	37 (37%)	
Consolidation therapy, yes	57 (57%)	
Number of consolidation therapies	57 (57%)	
Hematopoietic stem cell transplantation (HSCT), yes	30 (30%)	
Treatment outcomes		
Early death (ED), yes	19 (19%)	
Day of ED		17 (3–46)
Cause of ED		
Sepsis	10 (52.6%)	
Intracranial haemorrhage	4 (21.1%)	
Disseminated intravascular coagulation	2 (10.5%)	
Acute respiratory distress syndrome	1 (5.3%)	
Acute coronary syndrome	1 (5.3%)	
Neutropenic enterocolitis	1 (5.3%)	
Complete remission (CR), yes	57 (57%)	
Days to CR		48 (21–246)
Number of therapies needed for CR: 1/2/3	41 (72%), 13 (23%), 3 (5%)	
Primary refractory, yes	17 (17%)	
Measurable residual disease, present/out of tested	14/35 (40%)	
Relapsed, yes	33 (58%)	
Time to relapse in months		32.3 (2–324)
Therapy after relapse		
Palliation/chemotherapy (Cht)/Cht + HSCT	10 (30%)/17 (52%), 6 (18%)	
Survival		
DFS (months)		7.3 (0.3–88.2)
OS (months)		9 (0.3–88.2)

In Table 3, gene variants were presented by genotype frequencies.

**Table 3:** Gene variants presented by genotype frequencies.

Gene <br>Genotype	Number (%) <br>n=100 (100%)
*GSTM1*	
Null	52
Non-null	48
*GSTT1*	
Null	25
Non-null	75
*ABCB1*	
GG	36
GT	37
GA	1
TA	3
TT	23
*DCK*	
AA	15
AG	40
GG	45
*SLC29A1*	
CC	54
CT	39
TT	7

Demographic, standard laboratory and AML-related parameters were not predictive for CR, relapse, refractory disease, ED, DFS and OS. According to ELN 2022, the CR rate was 93.8% for favourable compared to 51.7% for the intermediate and 45.8% for unfavourable risk group (P=0.005). With respect to ELN 2022, lower odds for achieving a CR were in the intermediate (OR 0.071, 95% CI 0.009–0.574, P=0.013) and adverse (OR 0.056, 95% CI 0.006–0.498, P=0.01) groups, compared to favourable-risk group. None of the gene variants individually, tested in the dominant, recessive, and codominant manner, influenced either CR, relapse, ED, refractoriness, DFS, or OS (Table 4 and Table 5).

**Table 4:** Gene variants and treatment outcomes. Abbreviations: Co, codominant; Do, dominant; Re, recessive; CR, complete response; ED, early death; Σ, total.

Gene	Genotype	CR				ED				Refractory disease				Relapse			
Genetic model		Yes (%)	No (%)	(%)	p	Yes (%)	No (%)	(%)	p	Yes (%)	No (%)	(%)	p	Yes (%)	No (%)	(%)	p
*GSTM1*																	
Co	Null	30 (53)	22 (51)	52 (52)	0.884	10 (53)	42(52)	52(52)	0.951	10 (59)	42 (51)	52 (52)	0.536	20 (61)	32 (48)	52 (52)	0.227
	Non-null	27 (47)	21 (49)	48 (48)		9 (47)	39(48)	48(48)		7 (41)	41 (49)	48 (48)		13 (39)	35 (52)	48 (48)	
*GSTT1*																	
Co	Null	11 (19)	14 (33)	25 (25)	0.130	6 (32)	19 (23)	25 (25)	0.557	7 (41)	18 (22)	25 (25)	0.123	8 (24)	17 (25)	25 (25)	0.902
	Non-null	46 (81)	29 (67)	75 (75)		13 (68)	62 (77)	75 (75)		10 (59)	65 (78)	75 (75)		25 (76)	50 (75)	75 (75)	
*GSTM1, GSTT1*																	
Co	Double	6 (11)	4 (9)	10 (10)	1.000	0(0)	10 (12)	10 (10)	0.201	4 (23)	6 (7)	10 (10)	0.064	5 (15)	5 (8)	10 (10)	0.291
	Non dou-	51 (89)	39 (91)	90 (90)		19	71 (88)	90 (90)		13 (77)	77 (93)	90 (90)		28 (85)	62 (92)	90 (90)	
ABCB1																	
Do	GG	23 (40)	13 (30)	36 (36)	0.297	4 (21)	32 (40)	36 (36)	0.132	6(35)	30 (36)	36 (36)	0.947	12 (36)	24 (36)	36 (36)	0.958
	GT+GA+	34 (60)	30 (70)	64 (64)		15 (79)	49 (60)	64 (64)		11 (65)	53 (64)	64 (64)		21 (64)	43 (64)	64 (64)	
DCK																	
Co	AA	11 (20)	4 (9)	15 (15)	0.321	2 (10)	13 (16)	15 (15)	0.450	1(6)	14 (17)	15 (15)	0.489	8 (24)	7 (10)	15 (15)	0.191
	AG	23 (40)	17 (40)	40 (40)		10 (53)	30 (37)	40 (40)		7 (41)	33 (40)	40 (40)		12 (36)	28 (42)	40 (40)	
	GG	23 (40)	22 (51)	45 (45)		7 (37)	38 (47)	45 (45)		9 (53)	36 (43)	45 (45)		13 (40)	32 (48)	45 (45)	
Do	AA	11 (19)	4 (9)	15 (15)	0.166	2 (11)	13 (16)	15 (15)	0.729	1(6)	14 (17)	15 (15)	0,456	8 (24)	7 (10)	15 (15)	0.081
	AG+GG	46 (81)	39 (91)	85 (85)		17 (89)	68 (84)	85 (85)		16 (94)	69 (83)	85 (85)		25 (76)	60 (90)	85 (85)	
Re	GG	23 (40)	22 (51)	45 (45)	0.282	7 (37)	38 (47)	45 (45)	0.427	9 (53)	36 (43)	45 (45)	0,470	13 (39)	32 (48)	45 (45)	0.429
	AA+AG	34 (60)	21 (49)	55 (55)		12 (63)	43 (53)	55 (55)		8 (47)	47 (57)	55 (55)		20 (61)	35 (52)	55 (55)	
*SLC29A1*																	
Co	CC	31 (54)	23 (53)	54 (54)	1.000	10 (53)	44 (54)	54 (54)	0.231	9 (53)	45 (54)	54 (54)	1.000	17 (52)	37 (55)	54 (54)	0.501
	CT	22 (39)	17 (40)	39 (39)		6 (31)	33 (41)	39 (39)		7 (41)	32 (39)	39 (39)		15 (45)	24 (36)	39 (39)	
	TT	4 (7)	3 (7)	7 (7)		3 (16)	4 (5)	7 (7)		1 (6)	6 (7)	7 (7)		1 (3)	6 (9)	7 (7)	
Do	CC	31 (54)	23 (53)	54 (54)	0.929	10 (53)	44 (54)	54 (54)	0.894	9 (53)	45 (54)	54 (54)	0.923	17 (52)	37 (55)	54 (54)	0.726
	CT+TT	26 (46)	20 (47)	46 (46)		9 (47)	37 (46)	46 (46)		8 (47)	38 (46)	46 (46)		16 (48)	30 (45)	46 (46)	
Rec	TT	4 (7)	3 (7)	7 (7)	1.000	3 (16)	4 (5)	7 (7)	0.124	1 (6)	6 (7)	7 (7)	1.000	1 (3)	6 (10)	7 (7)	0.420
	CC+CT	53 (93)	40 (93)	93 (93)		16 (84)	77 (95)	93 (93)		16 (94)	77 (93)	93 (3)		32 (97)	61 (90)	93 (93	

**Table 5:** Gene variants and survival. Abbreviations: Co, codominant; Do, dominant; Re, recessive; OS, overall survival; DFS, disease-free survival; CI, confidence interval.

Gene	Genotype	DFS			OS		
Genetic model		Time (months)	95% CI	p	Time (months)	95% CI	p
*GSTM1*							
Co	Null	12.6	6.8–18.4	0.608	8.7	0.4–16.9	0.925
	Non-null	11.4	0–24.0		6.5	0.0–14.2	
GSTT1							
Co	Null	23.6	7.1–40.1	0.836	6	0.3–11.7	0.293
	Non-null	11.4	5.7–17.1		12.1	4.2–20	
*GSTM1, GSTT1*							
Co	Double null	24.1	11.5–36.7	0.399	7.6	2.8–14.6	0.824
	Non-double null	10.7	4.9–16.5		8.7	0–19.2	
*ABCB1*							
Do	GG	9.9	4.5–15.3	0.982	7	0.9–13	0.998
	GT+GA+TA+TT	14.6	5.1–24.1		9.3	0.9–17.6	
*DCK*							
Co	AA	22.5	18.9–26.1	0.551	21.6	12.0–31.2	0.615
	AG	20	5.3–34.6		6.0	0–19.8	
	GG	8.9	5–12.8		7.0	3.8–10.1	
Do	AA	22.5	18.9–26.1	0.526	21.6	12–31.2	0.379
	AG+GG	10.7	5.7–15.7		7	3.9–10.1	
Re	GG	8.9	5–12.8	0.286	7	3.8–10.1	0.447
	AA+AG	20.6	15.5–25.7		16.0	0–32.2	
*SLC29A1*							
Co	CC	18.3	6.9–29.7	0.365	10.4	2.6–18.2	0.646
	CT	11.4	7.5–15.2		9.3	0.0–19.3	
	TT	2.5	0.7–4.3		2.5	0.7–4.3	
Do	CC	18.3	6.9–29.6	0.510	10.4	2.6–18.2	0.534
	CT+TT	10.7	4.1–17.3		8.8	2.9–13.1	
Rec	TT	2.5	0.7–4.3	0.168	2.5	0.7–4.3	0.400
	CC+CT	14.6	5.3–23.9		9.7	3.5–16.1	

Besides, the combined effects of SLC29A1 rs9394992, DCK rs12648166, ABCB1 rs2032582 in dominant and SLC29A1 rs9394992 and DCK rs12648166 in a recessive manner showed no impact on clinical outcome and survival (Table 6 and Table 7). Of note is that ABCB1 was tested only in a dominant manner due to low frequencies of A allele in our group of patients (3 in TA and 1 in GA genotype).

**Table 6:** Combined effect of genotypes on clinical outcomes. Abbreviations: N, number; CR, complete response; ED, early death; Σ, total. <br>^a^ *SLC29A1* *CC, DCK AA, ABCB1 GG* genotype <br>^b^ *SLC29A1 TT, DCK GG* genotype

Genetic <br>model	Genotypes <br>(N)	CR				ED				Refractory disease				Relapse			
		Yes	No	Σ (%)	p	Yes	No	Σ (%)	p	Yes	No	Σ (%)	p	Yes	No	Σ (%)	p
Dominant^a^																	
	0	12 <brr>(21)	14 <br>(33)	26<br>(26)	0.163	6 <br>(32)	20 <br>(25)	26 <br>(26)	0.209	5<br>(29)	21 <br>(25)	26 <brr>(26)	0.256	7 <br>(21)	19 <br>(28)	26 <br>(26)	0.442
	1	26 <br>(45)	19 <br>(44)	45 <br>(45)		10 <br>(52)	35 <br>(43)	45 <br>(45)		8<br>(47)	37 <br>(45)	45 <br>(45)		15 <br>(46)	30 <br>(45)	45 <br>(45)	
	2	18 <br>(32)	9 <br>(21)	27 <br>(27)		3 <br>(16)	24 <br>(30)	27 <br>(27)		4 <br>(24)	23 <br>(28)	27 <br>(27)		11 <br>(33)	16 <br>(24)	27 <br>(27)	
	3	1 <br>(2)	1 <br>(2)	2 <br>(2)		0 <br>(0)	2 <br>(2.5)	2 <br>(2)		0 <br>(0)	2 <br>(2)	2 <br>(2)		0 <br>(0)	2 <br>(3)	2 <br>(2)	
Recessive^b^																	
	0	25 <br>(44)	18 <br>(42)	43 <br>(43)	0.836	8 <br>(42)	35 <br>(43)	43 <br>(43)	0.962	8 <br>(47)	35 <br>(42)	43 <br>(43)	0.988	15 <br>(46)	28 <br>(42)	43 <br>(43)	0.803
	1	22 <br>(39)	17 <br>(39)	39 <br>(39)		8 <br>(42)	31 <br>(38)	39 <br>(39)		5 <br>(29)	34 <br>(41)	39 <br>(39)		12 <br>(36)	27 <br>(40)	39 <br>(39)	
	2	10 <br>(17)	8 <br>(19)	18 <br>(18)		3 <br>(16)	15 <br>(19)	18 <br>(18)		4 <br>(24)	14 <br>(17)	18 <br>(18)		6 (18)	12 <br>(1)	18 <br<(18)	

**Table 7:** Combined effect of genotypes on survival. Abbreviations: N, number; OS, overall survival; DFS; disease-free survival; HR, hazard ratio; CI; confidence interval. <br>^a^SLC29A1 CC, DCK AA, ABCB1 GG genotype <br>^a^ SLC29A1 TT, DCK GG genotype

Genetic model	Genotypes (N)	OS			DFS		
		HR	95%CI	p	HR	95%CI	p
Dominant^a^	0	reference			reference		
	1	0.977	0.565–1.688	0.933	1.119	0.610–2.054	0.716
	2	0.812	0.436–1.511	0.510	0.807	0.403–1.614	0.544
	3	0.543	0.073–4.062	0.552	0.685	0.091–5.175	0.714
Recessive^b^	0	reference			reference		
	1	1.321	0.805–2.168	0.271	1.651	0.953–2.861	0.074
	2	1.103	0.586–2.074	0.762	1.271	0.619–2.610	0.514

Furthermore, all of the independent predictors at the significance level 0.05 in the univariate analysis were included in the multivariate COX regression model with OS (age >55, ELN risk categories and CR) and DFS (age >55 and CR) as dependent variables. Due to multicollinearity with the CR variable, the model did not include primary refractoriness, consolidation, and HSCT. Due to only two CD25-positive cases and the fact that all patients were not treated after the relapse, these two parameters were not included in the model. The multivariate model revealed CR as an independent predictor for DFS (HR 0.284, 95%CI=0.158–0.511, P=<0.01) and OS (HR 0.114, 95%CI=0.064–0.204, P=<0.01).

## Discussion

Despite the high CR rate after induction of ChT, the main reason for relapse is the ineffectiveness of ChT to eliminate MRD [5]. Moreover, some patients die because of ChT toxicity, primary resistant disease, or relapse [5]. So far, the best pre-treatment predictors of outcome in AML patients are the cytogenetic/ molecular aberrations [1]. In our study, we analysed the effect of ELN 2022 cytogenetic classification [1] that was only predictive of the CR rate while failing to predict other clinical outcomes in a group of patients. Moreover, none of the demographic, standard laboratory, or AML-related parameters predicted the disease outcome. Multivariate analysis identified CR as an independent prognostic factor for DFS and OS, while age >55 years and ELN 2022 risk stratification and gene variants failed to demonstrate an impact on clinical outcomes in our group of AML patients.

Human equilibrative nucleoside transporter 1 (hENT1) encoded by the *SLC29A1* gene is a primary influx transporter, responsible for the transport of ~80% of cytarabine into a leukemic cell, especially during induction courses (100–200 mg/m^2^) when plasma concentrations of cytarabine are <1 μmol/L [6]. The expression of hENT1 is correlated with clinical outcomes [19], whereas low mRNA expression is associated with DFS and OS in adult AML [20]. In our study, variant rs9394992 of *SLC29A1* showed no relation to clinical outcome, which is in line with Japanese [21] and Korean [22] studies. A higher CR rate was observed in haplotype ht3 (including the T allele of rs9394992) [22]. In contrast, in the cohort of Chinese patients, a lower relapse rate and longer OS and DFS were associated with the CC genotype, compared to CT/TT [15].

The first and rate-limiting step of cytarabine activation into cytarabine-triphosphate, an active antileukemic form of cytarabine, is mediated by *DCK*. Previous studies on higher pre-treatment mRNA expression of *DCK* are related to longer event-free survival in AML patients treated with cytarabine and in solid malignancies treated with gemcitabine, another nucleoside analogue [23] [24]. Our results are in line with Japanese [21] and Chinese studies [15], which showed no influence of the DCK rs12648166 variant on outcomes in Asian AML patients.

Drug resistance to standard ChT in AML (Multi Drug Resistance, MDR) is genetically determined [25]. One of the main mechanisms of MDR is transport (pump) resistance, which is represented by the increased expression of drug efflux pumps [26] [27]. One of the most evaluated is *MDR1* (p-glycoprotein), an anthracycline efflux pump encoded by *ABCB1* gene. Lower pump function, thus higher intracellular anthracycline concentration, was correlated to higher CR and OS and higher toxicity [6]. This finding is confirmed in the previous studies [28] [29] [30] [31] and in two metanalyses [32] [33] that evaluated variants of *ABCB1*, including the rs2032582 (2677G>T/A). Contrary to these findings, in our group of patients rs2032582 variant of *ABCB1* showed no influence on clinical outcomes in AML patients. This finding corresponds with previous studies in Germans [34], Turks [35], Dutch [36] patients from the United States of America [37], Swedish [38], Spanish [39], and South Koreans [28] [29].

Gene variations in GST, the main intracellular detoxifiers of ROS induced by anthracyclines, are associated with clinical outcomes in AML. Namely, a meta-analysis of 11 studies covering 1837 patients (ranging from 63 to 353 patients in each study) revealed that deleterious (null) genotypes of *GSTT1* and double null genotypes of *GSTT1* and *GSTM1* are related to the reduced CR rate, progression-free survival, and OS, especially in the Asian population [40]. Only one study on 106 Italian AML patients (86% newly diagnosed) [41], out of seven studies evaluated in Caucasians [5], showed a lower CR rate, EFS, and OS in null genotypes of *GSTT1* or *GSTM1*. We have not confirmed this correlation in our group of 100 Serbian patients. Of note is that in our group, the double null genotype of *GSTT1* and *GSTM1*, was close to statistical significance (p=0.06) for primary refractoriness. Given that the null genotype of *GSTT1* was more frequent in the Asian population [42], the more prominent effect of these deletions on prognosis in AML could be partially explained in this population.

Combined effects of other ABC and *SLC* gene variants were explored in earlier studies with findings of increased toxicity and higher ED rate [6]. Besides, the combined effects of rs9394992 and rs324148 of *SLC29A1* [15] and the combined effects of different *SLC29A1* variants with variants in other genes of the cytarabine metabolic pathway [22] [43] showed an impact on clinical outcomes in AML patients. In our study, the combined effect of tested variants *SLC29A1, DCK*, and *ABCB1* did not influence the clinical outcome.

Most of the studies that showed the impact of gene variants on clinical outcomes in AML patients were conducted in the Asian population [44]. These results are not confirmed in our study group. One possible explanation could be that the different genotype frequencies between Asian and Serbian populations diminished the impact of these gene variants on clinical outcomes. This explanation referred mainly to the *SLC29A1, GSTT1*, and *GSTM1* gene variants, which primarily impacted clinical outcomes in the Asian population. The lack of influence of *ABCB1* variants on AML prognosis could be explained by the low frequency of minor allele A in our group of patients.

Furthermore, highly variable treatment patterns used in the studies regarding drug dosage (cytarabine 100–200 mg/m^2^, daunorubicin 45–90 mg/m^2^), use of other anthracyclines (e.g., idarubicin) or the use of other drugs in addition to cytarabine and anthracycline (e.g., etoposide, amsacrine, fludarabine) could have influenced the clinical outcomes in relation to these gene variants. Besides, demographic and AML-related variables in different proportions across the studied groups could also impact the outcome of AML patients.

The inconsistency in the studies on AML pharmacogenetics can be partially explained by the existence of a population-specific pharmacogenomic profile, demonstrated in numerous studies [45]. Data on AML pharmacogenomics in the European population are lacking. Therefore, comprehensive studies must be conducted to get data on reliable AML pharmacogenetic markers for European populations. The first results for the Serbian population contribute to overcoming the knowledge gap in this field. Furthermore, high-throughput analysis, even a multi-omics approach, is mandatory to determine clinically actionable pharmacogenomic/pharmacomic markers. Modern medicine will only provide personalized treatment for each AML patient.

## Conclusion

Clinical outcomes in our sample of AML patients were not impacted by variants of *SLC29A1, DCK, ABCB1* and *GSTT1* and *GSTM1*, independently or in combinations. Only achievement of CR was identified as an independent prognostic indicator of clinical outcome in AML patients.

The population-specific genomic profile has to be considered in pharmacogenetics. Since the data on AML pharmacogenetics in European populations is limited, our results contribute to knowledge in this field and strongly indicate that a high-throughput approach must be applied to find particular pharmacogenetic markers of AML in Serbian and European populations.

## Dodatak

### Acknowledgements

This study was supported by the Ministry of Education, Science and Technological Development of Serbia, Project N^0^ III41004, and PharmGenHUB project funded by EU HORIZON EUROPE, grant agreement N^0^ 101059870.

### Conflict of interest statement

All the authors declare that they have no conflict of interest in this work.
